# Historical Roles of Selenium and Selenoproteins in Health and Development: The Good, the Bad and the Ugly

**DOI:** 10.3390/ijms23010005

**Published:** 2021-12-21

**Authors:** Petra A. Tsuji, Didac Santesmasses, Byeong J. Lee, Vadim N. Gladyshev, Dolph L. Hatfield

**Affiliations:** 1 Department of Biological Sciences, Towson University, 8000 York Rd., Towson, MD 21252, USA; 2 Brigham and Women’s Hospital, Harvard Medical School, Boston, MA 02215, USA; dsantesmassesruiz@bwh.harvard.edu (D.S.); vgladyshev@rics.bwh.harvard.edu (V.N.G.); 3 School of Biological Sciences, College of Natural Sciences, Seoul National University, Seoul 08826, Korea; imbglmg@snu.ac.kr; 4 Scientist Emeritus, Mouse Cancer Genetics Program, Center for Cancer Research, National Cancer Institute, National Institutes of Health, Bethesda, MD 20892, USA; hatfielddolph@gmail.com

**Keywords:** cancer, health, mouse models, selenium, selenocysteine (Sec), tRNA[Ser]^Sec^, Sec-tRNA[Ser]^Sec^, selenoproteins

## Abstract

Selenium is a fascinating element that has a long history, most of which documents it as a deleterious element to health. In more recent years, selenium has been found to be an essential element in the diet of humans, all other mammals, and many other life forms. It has many health benefits that include, for example, roles in preventing heart disease and certain forms of cancer, slowing AIDS progression in HIV patients, supporting male reproduction, inhibiting viral expression, and boosting the immune system, and it also plays essential roles in mammalian development. Elucidating the molecular biology of selenium over the past 40 years generated an entirely new field of science which encompassed the many novel features of selenium. These features were (1) how this element makes its way into protein as the 21st amino acid in the genetic code, selenocysteine (Sec); (2) the vast amount of machinery dedicated to synthesizing Sec uniquely on its tRNA; (3) the incorporation of Sec into protein; and (4) the roles of the resulting Sec-containing proteins (selenoproteins) in health and development. One of the research areas receiving the most attention regarding selenium in health has been its role in cancer prevention, but further research has also exposed the role of this element as a facilitator of various maladies, including cancer.

## 1. Introduction

The element selenium was discovered in 1817 by the Swedish chemist, Jöns Jacob Berzelius [1]. He named selenium after the Greek goddess of the moon, Selene. This fascinating element has a long and unsavory history of use as a dietary component. Its first description as being deleterious for animals to ingest was reported by Marco Polo in the late 13th century [2]. In his travels in Western China, Marco Polo wrote about an illness that his “beasts of burden” acquired wherein their hooves became brittle and fell off after eating certain plants. These plants most likely were seleniferous plants, which absorb large quantities of selenium from the soil and store the selenium in their tissues. Such diseases as Polo described have been found in the 20th century in horses and cattle that grazed on the plains of the Dakota and Nebraska territories of the United States. For example, T.C. Madison, an army physician stationed at Fort Randall in Northern Nebraska in the mid-1850s, described a necrotic hoof disorder that, in addition, involved losses of hair in the mane and tail among the army horses that grazed on the plants around the fort [3].

Subsequently, Franke reported that this malady, which was found to be prevalent in the livestock living in these Great Plains states, resulted from these animals eating seleniferous plants that were rich in selenium absorbed from high levels of this element in the soil [4]. Interestingly, and to further document the harmful effects of selenium on the animals ingesting plants rich in this element, selenium poisoning in horses was thought to have played a role in General George Custer’s defeat at the Little Bighorn [5]. The military horses under Custer’s command had grazed freely on the plants surrounding the area where he and his men had waited prior to the battle; these plants were later found to be seleniferous plants. At the Battle of the Little Bighorn on 25 June 1876, Custer’s horses were reported to have had laminitis, which caused them to be lame, and led to Custer’s defeat [5].

Selenium’s role as a deleterious dietary element took a major turn for the good in 1957, when Schwarz and Foltz unexpectedly found that it prevented liver necrosis in rats [6]. The Schwartz and Foltz finding was followed by another interesting observation that selenium had an important role in anaerobic growth in *Escherichia coli* when this organism was grown on glucose [7]. It soon became obvious that selenium was an essential element in the diet of mammals and many other life forms when ingested in low levels, but harmful when ingested in high levels (see several chapters in [8] for an in-depth summary on these findings). The window between too little and too much selenium in the diet is rather narrow for most organisms.

Subsequently, the livestock industry found that the inclusion of selenium in the diet of livestock had many health benefits. These included enhanced fertility in male sheep and cattle, and importantly also the alleviation of numerous disorders such as white muscle disease and ill thrift in lambs and calves, pancreatic degeneration and exudative diathesis in fowl, and *hepatosis dietetica* in swine [9]. In many regions of the world where livestock are prevalent, the addition of selenium in the diets of livestock has saved this industry hundreds of millions of dollars [10]. With regard to humans, in certain regions in rural China, where the soil is deficient in selenium and hence the selenium status of the individuals living therein is suboptimal, maladies such as Keshan disease, a cardiomyopathy primarily in children, were found [11]. Similarly, Kashin–Beck disease, a chronic, endemic osteochondropathy, was found primarily in southwestern to northeastern China [12]. Keshan disease has been virtually eradicated in China by supplementing the diets of the populations residing in specific rural areas where the soil is deficient in selenium [13]. In the USA, the recommended daily amount of selenium is set forth by the Food and Nutrition Board of the National Academies of Medicine, and is 55 micrograms per day for men and women above 14 years of age. Women who are pregnant or lactating require 60 or 70 micrograms per day [14,15].

In addition to having roles in preventing heart disease and other muscle disorders, as well as enhancing male fertility, selenium was found to have roles as a chemopreventive agent in certain cancers [16,17,18,19], roles in boosting immune function [18,20], in suppressing viral expression [21], in slowing the development of AIDS in HIV positive patients [22] and in Simian Acquired Immunodeficiency Virus (SAIDS)-infected monkeys [23], and possibly in slowing the aging process [24].

Low molecular weight selenium-containing compounds (LMW selenocompounds) also have highly significant roles in providing benefits to mammals. The research carried out in this area constitutes a subfield within the selenium field. There are several excellent reviews on the benefits of LMW selenocompounds in health and numerous other aspects of these selenocompounds [25,26,27]. This topic will, therefore, not be further discussed herein.

Several seminal studies in the selenium field in the 1970s and 1980s provided the foundation for elucidating the molecular biology of selenium and established it as a separate and highly significant field in science. Initially, selenium was found to be an essential component of glutathione peroxidase 1 (GPX1) in 1973 [28,29], which was subsequently identified in clostridial glycine reductase as selenocysteine (Sec) [30]. Bovine GPX1 was sequenced in 1984, and the position of the Sec moiety was therefore established within the protein [31]. The gene sequences of mammalian *Gpx1* [32] and bacterial glycine formate dehydrogenase [33] were determined, and the Sec residue in the corresponding proteins shown to be encoded by TGA in both genes.

Additional studies that played major roles in defining the molecular biology of selenium as a separate field of science rapidly developed, and encompassed how selenium made its way into protein as the 21st proteinogenic amino acid in the genetic code—the vast machinery dedicated to synthesizing Sec and incorporating it into protein—and the roles of the resulting Sec-containing proteins (selenoproteins) in health and development. One of the research areas receiving much attention has been the role of selenium in cancer prevention, but this finding has also exposed the potential role of this element as a facilitator of various maladies, including cancer. These aspects of the molecular biology of selenium are discussed herein.

## 2. Selenocysteine (Sec) tRNA[Ser]^Sec^

This Section describes various aspects of Sec tRNA[Ser]^Sec^ (i.e., the transcription of tRNA[Ser]^Sec^, primary sequences of the two isoforms, their distributions, the synthesis of Sec on tRNA[Ser]^Sec^, and the incorporation of Sec into selenoproteins as the 21st amino acid in the genetic code). The reason Sec tRNA is designated tRNA[Ser]^Sec^ is that it is initially aminoacylated with serine (Ser) by Ser-tRNA synthetase (SARS), and the Ser moiety is then uniquely converted to Sec on the tRNA (see Section 2.4 below).

### 2.1. Transcription of the tRNA[Ser]^Sec^ Gene (Trsp)

*Trsp* is a single-copy gene in most genomes, but several organisms, including zebrafish, have more than one copy [34,35]. Transfer RNA[Ser]^Sec^ is transcribed, like all canonical tRNAs, by RNA polymerase (Pol) III, except in *Trypanosoma brucei* which was reported to be transcribed by Pol II [36]. However, the promoter structure and TATA-box-binding protein utilization of tRNA[Ser]^Sec^ are distinct from those of other tRNA genes [37,38]. While the transcription of canonical tRNA genes is dependent on the internal promoters, so called A- and B-boxes, the upstream promoters including TATA-boxes govern the transcription of tRNA[Ser]^Sec^ and other TATA-less Pol III genes such as snU6 and 7SK. *Trsp* transcription is initiated at the first nucleotide within the gene, whereas all other tRNAs are transcribed with a leader sequence that must be removed by processing from the resulting transcript [39]. The tRNA[Ser]^Sec^ transcript has a trailer sequence, and like all other tRNAs, the trailer must be processed to yield the primary sequence, wherein the ubiquitous CCA terminus is then added to prepare the completed transcript which is now ready for modification.

### 2.2. Primary Sequence of Sec tRNA[Ser]^Sec^

**Figure 1:**
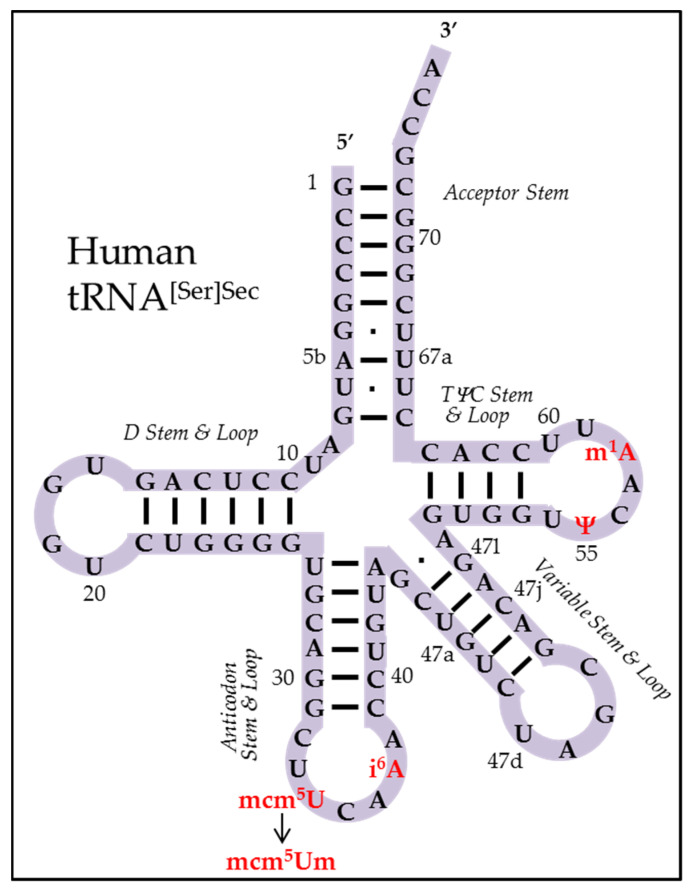
Cloverleaf model of human tRNA[Ser]^Sec^. The image shows the 90 bases in human tRNA[Ser]^Sec^. The paired 5′ and 3′ terminal bases constitute the acceptor stem, and on the left portion of the tRNA, the D stem and loop constitute six paired and four unpaired bases. On the lower portion of the tRNA, the anticodon stem and loop constitute six paired and seven unpaired bases, and the variable stem and loop constitute five paired and four unpaired bases. On the right portion of the tRNA, the TΨC stem and loop constitute four paired and seven unpaired bases. Human tRNA[Ser]^Sec^ contains base modifications at the following positions: 34 (mcm^5^U), 37 (i^6^A), 55 (Ψ), and 58 (m^1^A). The two isoforms differ from one another at position 34 by a single methyl group on the 2′-O-ribosyl moiety.

The primary sequence of Sec tRNA[Ser]^Sec^, which is the longest tRNA described to date, contains 90 or more nucleotides, depending on the species that encodes Trsp. In higher animals (e.g., *Xenopus*, birds, and mammals), four bases are modified on the 90 nucleotide primary transcript, and a portion of the Sec tRNA population is modified on the 2′-O-ribosyl moiety forming the only nucleoside modification (reviewed in [40]). The four base modifications are 5-methoxycarbonylmethyluracil (mcm^5^U) at position 34 (the wobble position in the anticodon), isopentenyladenosine (i^6^A) at position 37 (the base immediately 5′ to the anticodon), pseudouridine (Ψ) at position 55, and N1-methyladenosine (m1A) at position 58 (wherein the last two modifications occur within the TΨC loop). The single nucleoside modification occurs when a portion of the mcm^5^U isoform is converted to 5-methoxycarbonylmethyluracil-2′-O-methylribose (mcm^5^Um). The methylation of mcm^5^U to mcm^5^Um requires other modifications such as i^6^A and m^1^A, and the correct tertiary structure [41]. Interestingly, methylation of mcm^5^U is influenced by the selenium levels in the cell [41]. The primary structures of tRNA[Ser]^Sec^_mm_^5^_U_ and tRNA[Ser]^Sec^_mm_^5^_Um_ are shown in a cloverleaf form in Figure 1.

### 2.3. The Sec-tRNA[Ser]^Sec^ Population

The Sec-tRNA[Ser]^Sec^ populations in bacteria and archaea consist of a single tRNA that is aminoacylated with Ser by seryl-tRNA synthetase (SARS). The tRNA[Ser]^Sec^ populations in mammals, birds, and Xenopus consist of two isoforms: tRNA[Ser]^Sec^_mcm_^5^_U_ and tRNA[Ser]^Sec^_mm_^5^_Um_, both of which are aminoacylated with Ser by their corresponding SARS [18]. The levels of the two isoforms are not limiting; however, reducing the tRNA[Ser]^Sec^ population by about half or increasing it several-fold does not generally appear to affect overall selenoprotein expression in various mammalian cells and mouse tissues [42]—albeit some minor differences in selenoprotein expression have been observed [43].

The levels of mcm^5^U are enriched and those of mcm^5^Um diminished under conditions of selenium deficiency in mammalian cells and organs, while the reverse is true under conditions of selenium sufficiency, i.e., the levels of mcm^5^Um are enriched, and the levels of mcm^5^U diminished. Interestingly, the two Sec-tRNA[Ser]^Sec^ isoforms are involved in the synthesis of different classes of selenoproteins. Housekeeping selenoproteins, such as GPX4 and thioredoxin reductase 1 (TXNRD1), are essential to cellular function and are expressed even during selenium-deficient conditions. Housekeeping selenoproteins are expressed by the Sec-tRNA[Ser]^Sec^_mcm_^5^U isoform. Stress-related selenoproteins, such as GPX1, are expressed in higher amounts in response to enriched selenium levels. This class of selenoproteins is expressed by the Sec-tRNA[Ser]^Sec^_mcm5Um_ isoform [43,44].

Detailed examinations of Ser-tRNA[Ser]^Sec^_mcm_^5^U and Ser-tRNA[Ser]^Sec^_mcm_^5^Um levels were carried out in various mammalian cell lines by growing human leukemia (HL-60) cells, Chinese hamster ovary (CHO) cells, and rat mammary tumor (RMT) cells in media with or without selenium supplementation (Table 1), and in various mammalian organs by subjecting mice to diets with or without selenium supplementation (Table 2). The respective tRNA populations were isolated from each cell line and from each mouse organ, labeled with ^3^H-serine, and the distributions of the two Sec isoforms were determined. The total amount of the two Ser-tRNA[Ser]^Sec]^ isoforms varied considerably in the different cell lines and mammalian organs (Table 1 and Table 2), respectively. The consistent observation was that in every case the tRNA[Ser]^Sec^_mcm_^5^U isoform was enriched in selenium-deficient conditions and the Ser-tRNA[Ser]^Sec^_mcm_^5^Um isoform was enriched in selenium-sufficient conditions.

### 2.4. Biosynthesis of Sec on Sec tRNA[Ser]^Sec^

**Figure 2:**
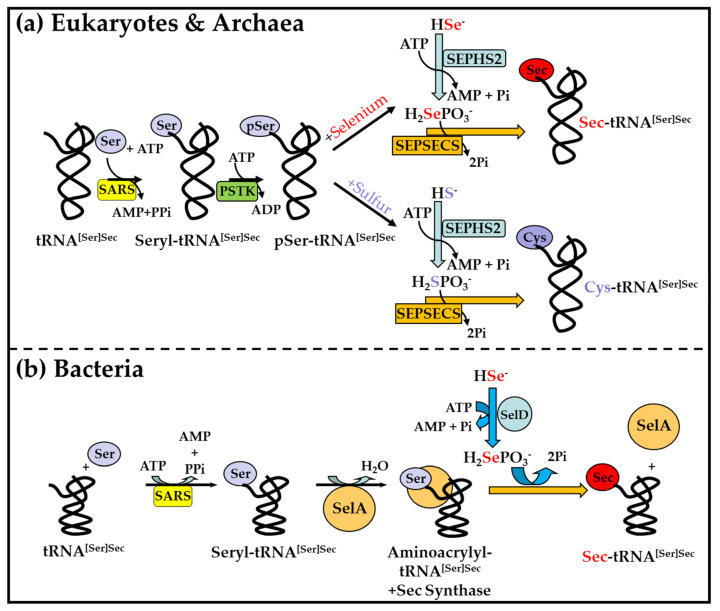
Pathways of Selenocysteine (Sec) biosynthesis. The biosynthetic pathways of Sec in: (**a**) eukaryotes and archaea; (**b**) bacteria. Abbreviations are: Pi—inorganic phosphate; PPi—inorganic pyrophosphate; SARS—Ser-tRNA synthetase; SelA—selenocysteine synthase; SelD—selenophosphate synthetase; H_2_SePO_3_^−^—selenophosphate; SEPHS2—selenophosphate synthetase 2.

The incorporation of selenium into protein occurs as the amino acid Sec. This amino acid is biosynthesized in a unique manner on its tRNA, named Sec-tRNA[Ser]^Sec^ [45,46]. The pathway of Sec biosynthesis is different in archaea and eukaryotes (Figure 2a), and in bacteria (Figure 2b). The unacylated tRNA[Ser]^Sec^ is initially aminoacylated with Ser by SARS to form Ser-tRNA[Ser]^Sec^ in all three groups. The Ser moiety on Ser-tRNA[Ser]^Sec^ in archaea and eukaryotes (Figure 2a) is transferred to an intermediate, phosphorseryl-tRNA[Ser]^Sec^, by phosphoseryl-tRNA kinase (PSTK). The intermediate is then converted to Sec-tRNA[Ser]^Sec^ in the presence of selenophosphate 2 (SEPHS2) (see [18] and references therein). On the other hand, bacteria use an enzyme, Sec synthetase (SecS or SepSecS), to convert Ser-tRNA[Ser]^Sec^ to Sec-tRNA[Ser]^Sec^ (Figure 2b). There is an abundance of complex machinery dedicated to incorporating Sec into protein in response to the UGA Sec codon in selenoprotein mRNA. This topic has been reviewed elsewhere in this Special Issue by Copeland and Howard [47], and, therefore, will not be further discussed herein. The insertion of Sec into protein to generate selenoproteins occurs in organisms within all three of the taxonomic domains, eukaryotes, archaea, and bacteria. Selenoproteins are found in only about 15% of archaea, about 25% of bacteria, and about half of eukaryotes [48].

### 2.5. Sec, the 21st Amino Acid in the Genetic Code

Sec constitutes the 21st proteinogenic amino acid in the genetic code, and Sec is encoded in selenoprotein mRNA, as noted above, by the genetic codeword UGA. UGA is a shared codon in the genetic code in organisms containing selenoproteins, and it designates either Sec or the cessation of protein synthesis depending on its location within the mRNA. A specific sequence called the Sec insertion sequence (SECIS) element, which is located immediately downstream of the Sec UGA codon in bacteria and located much further downstream of the Sec UGA codon in archaea and eukaryotes, is responsible for designating the upstream codon as Sec [49]. The classes of SECIS elements and their roles have been reviewed in detail elsewhere (see [18,45] and references therein) and will not be further considered herein.

## 3. Selenoproteins

### 3.1. Mammalian Selenoproteins

There are 25 selenoprotein genes in the human genome. They are highly conserved across mammals, with the only two known exceptions being GPX6 and SELENOV. GPX6 contains Cys instead of Sec in some species, including mice and rats, and SELENOV was lost in gorillas [50]. Some of the mammalian selenoproteins are shared with non-mammalian eukaryotes, including glutathione peroxidases (GPXs), thioredoxin reductases (TXNRDs), and selenophosphate synthetase (SEPHS), indicating an early evolutionary origin for these proteins [51].

#### 3.1.1. Glutathione Peroxidases (GPX)

GPXs comprise a large superfamily that is widespread across all kingdoms of life [52]. They use glutathione or thiol oxidoreductases as major reductants, and their functions include detoxification of hydroperoxides, regulation of ferroptosis, and hydrogen hydroperoxide signaling, among others [51]. There are eight GPXs in mammals, five of which are selenoproteins (GPX1-4, GPX6), and three are Cys-containing homologs (GPX5, GPX7, and GPX8). GPX1 is the most abundant mammalian selenoprotein found in the cytosol of most cells. It was the first mammalian selenoprotein described, and it was instrumental in developing methods for the insertion of selenium in the form of Sec into proteins. GPX2 and GPX3 have a more localized expression. GPX2 is often termed gastrointestinal based on its initial detection in gastrointestinal tissues, and GPX3 is expressed in the kidney at very high levels and is secreted into the plasma. GPX4 is unique among the GPXs because it reduces phospholipid hydroperoxides and has a broad substrate specificity. It has received much attention recently due to its essentiality for embryonic development in mice [53,54] and its role as a master regulator of ferroptosis [55,56]. GPX6 is the most recently evolved Sec-containing GPX, present only in mammals. In mice and in a few other species, Sec was then replaced by Cys [50].

#### 3.1.2. Thioredoxin Reductases (TXNRD)

There are three TXNRDs in mammals, all of which contain Sec, and their functions are selenium-dependent. Sec is located in the penultimate C-terminal position, as part of a characteristic GCUG motif [57]. TXNRD1 is primarily localized in the cytosol and nucleus, and uses thioredoxin 1 (TXN1) as a major substrate. Its main physiological role is the NADPH-dependent reduction of TXN1, which in turn is involved in many physiological processes. TXNRD2 is localized in the mitochondria, and it is involved in the reduction of mitochondrial thioredoxin (TXN2) and glutaredoxin 2 (GLRX2). Both TXNRD1 and TXNRD2 are present in all vertebrates and are essential in mice [58,59]. TXNRD3 contains an additional N-terminal GLRX domain, and displays glutaredoxin activity [60].

#### 3.1.3. Iodothyronine Deiodinases (DIO)

The thyroid hormone deiodinases (DIO) consist of three selenoproteins (DIO1, DIO2, and DIO3) that are involved in the metabolism of thyroid hormones by iodothyronine deiodination [61]. Like most selenoproteins, they are thioredoxin-like proteins. Thyroid hormones regulate a variety of processes, including growth, development, and metabolic rate. Thyroxine (T4) is the main thyroid hormone in circulation, produced in the thyroid gland, and is the precursor of triiodothyronine (T3), which has a higher affinity for thyroid hormone receptors [62]. DIO1 and DIO2 catalyze the activation of T4 to T3. Conversely, DIO3, and in some conditions DIO1, can inactivate T3 by producing the inactive metabolites T2 and reverse T3 (rT3), respectively. Studies in deiodinase-deficient mice have confirmed the function of deiodinases for T3 formation [63,64,65]. Distantly related homologs have been identified in single-celled eukaryotes; however, their function is not known, but it must be different from that of mammalian deiodinases.

#### 3.1.4. Methionine-R-Sulfoxide Reductase 1 (MSRB1)

MSRB1 is a zinc-containing selenoprotein that was initially identified as selenoprotein R [66] and selenoprotein X [67] using bioinformatic tools. It was later found to be methionine-R-sulfoxide reductase based on its repair activity on the R enantiomer of oxidized methionine residues in proteins. Methionine and cysteine are the two sulfur-containing amino acids that are the most susceptible to oxidation, which may lead to a significant alteration of their structure and the disruption of protein function. MSRB1 may protect proteins against oxidative damage by catalyzing the reduction of methionine sulfoxide back to methionine. Though structurally different to MSRA (methionine-S-sulfoxide reductase), both proteins perform complementary functions by acting in only one of the two stereoisomers. MSRA is also a selenoprotein in some unicellular eukaryotes. Two additional homologs, MSRB2 and MSRB3, which contain catalytic Cys instead of Sec, are present in mammals. MSRB2 is localized in the mitochondria, whereas MSRB3 is targeted to the endoplasmic reticulum [68].

#### 3.1.5. Selenophosphate Synthetase 2 (SEPHS2)

As discussed above, SEPHS2 provides the active Se donor for the synthesis of Sec. Selenophosphate is synthesized from selenide and ATP [69]. SEPHS2 is a widespread protein found in all Sec-containing prokaryotes and eukaryotes. In prokaryotes, in addition to Sec, SEPHS2 also supports other forms of selenium utilization. Selenium is used in the form of selenouridine in certain tRNAs [70,71], and as a cofactor in some molybdenum-containing proteins [72,73]. In eukaryotes, Sec is the only known selenium trait.

A SEPHS2 paralog called SEPHS1 is found in some animals [74,75]. SEPHS1 is not a selenoprotein, it does not support selenophosphate and selenoprotein synthesis [76], and never carries Sec or Cys at the catalytic site. Instead, other amino acids have been observed at that position (arginine, threonine, glycine, and leucine). SEPHS1 is an essential gene in fruit flies [77] and mice [78], but its function remains unknown. The fact that SEPHS1 is present in selenoprotein-less animals [79,80] suggests that its function is not related to selenium. Nonetheless, it is believed to participate in redox homeostasis [78]. Interestingly, SEPHS1 genes in different animal lineages, e.g., insects and vertebrates, are believed to be functional homologs, but they originated independently [81].

#### 3.1.6. Selenoprotein P (SELENOP)

SELENOP is the only selenoprotein with multiple Sec residues in mammals. It is a major selenoprotein in plasma and is synthesized primarily in the liver [82]. Its sequence contains a Sec-containing thioredoxin-like domain in its N-terminal region, and a Sec-rich domain in the C-terminus [83]. Its main function is to provide selenium to several tissues, especially the testis and brain [84,85]. SELENOP is present across metazoans, but its Sec content is highly variable. Among vertebrates, the number ranges from five in mole rats to up to 37 in amberjack fish. Human and mouse SELENOP contains 10 Sec residues. A remarkable diversity is observed in invertebrates. SELENOP was lost in most nematodes, most insects, tunicates, and Platyhelminthes, whereas in other lineages, SELENOP is particularly Sec-rich, including in annelids, ribbon worms (Nemertea), and mollusks. Topping the list is the bivalve *Elliptio complanata* with 133 Sec residues [86].

#### 3.1.7. Selenoprotein N (SELENON)

SELENON (formerly SEPN1) is an endoplasmic reticulum (ER) glycoprotein that contains a calcium-binding EF-hand domain and a Sec-containing domain with a possible oxidoreductase function. The specific function of the protein remains unknown. One possible function that has been suggested is a calcium sensor through the EF-hand domain, which activates the sarcoplasmic/ER calcium ATPase 2 (SERCA2)-mediated calcium uptake into the ER in a redox-dependent manner [87]. SELENON was first identified using computational methods [67], and shortly after was associated with congenital rigid spine muscular dystrophy [88], becoming the first selenoprotein to be associated with a genetic disease. Mutations in SELENON cause a group of recessive neuromuscular disorders collectively known as SELENON-related myopathies [89]. Many mutations have been identified in homozygous or heterozygous compound patients, including mutations in the UGA codon and the SECIS element that prevent the incorporation of Sec [88,90,91].

#### 3.1.8. Selenoprotein O (SELENOO)

SELENOO is a widespread selenoprotein present in both prokaryotes and eukaryotes. The mammalian homologs carry a Sec residue at their C-terminal penultimate position [92], while in bacteria and in many other eukaryotes, including yeast and plants, homologs have a Cys instead. Its sequence contains a protein kinase-like domain [93] but its function remained elusive [92]. A recent study [94] uncovered a novel activity for the protein kinase superfamily, wherein SELENOO transfers AMP from ATP to Ser, Thr, and Tyr residues on protein substrates, an activity termed AMPylation. The protein is localized in the mitochondria [95] and AMPylates proteins involved in redox homeostasis [94].

#### 3.1.9. Selenoprotein I (SELENOI)

SELENOI is essential for embryonic development in mice [96]. It is a recently evolved selenoprotein only found in vertebrates [50]. Its sequence contains a CDP-alcohol phosphatidyltransferase domain, characteristic of phospholipid synthases. It was suggested that SELENOI may be an ethanolamine phosphotransferase that catalyzes the last step of the Kennedy pathway for the synthesis of phosphatidylethanolamine [97] and is localized in the Golgi apparatus [98]. However, this activity was reported for a protein truncated at Sec, so more studies are needed to establish the function of this selenoprotein. Mutations in SELENOI have been identified in patients with a form of hereditary spastic paraplegia [99,100].

#### 3.1.10. Other Selenoproteins

Other selenoproteins have no known functions, albeit some suggested ones. SELENOW, SELENOT, SELENOH, and SELENOV belong to the redox family of selenoproteins [101]. They possess a CXXU motif within a thioredoxin-like fold domain. Based on this observation, they are proposed to be oxidoreductases of unknown functions. SELENOV is the most recently evolved selenoprotein, only present in placental mammals. It appeared by duplication of SELENOW; the two genes share the same exonic structure, but SELENOV contains a long highly variable N-terminal extension [50]. SELENOV is exclusively expressed in testis [92].

SELENOF and SELENOM are thioredoxin-like fold ER-resident selenoproteins. These proteins share ~30% of sequence identity in mammals and are distantly related homologs with a common evolutionary origin [51]. Their function, however, is not well understood. SELENOF may be involved in the regulation of protein folding, and its deletion promotes nuclear cataracts in mice [102]. Several studies examined its possible role in cancer [103,104]. Altered expression, both high and low, has been linked to a higher cancer risk in different tissues, including lung, breast, prostate, and liver [105]. Similarly, common genetic variants in SELENOF have been studied for their relationship with higher cancer risk [105]. SELENOM is expressed in the brain and shows neuroprotective properties. Altered levels of SELENOM have been linked to the early onset of Alzheimer’s disease and hepatocellular carcinoma [106]. In addition, it has also been implicated in calcium release from the ER in response to hydrogen peroxide [107]. Its deletion in mice leads to increased body weight but does not affect neuronal and cognitive function [108].

SELENOK and SELENOS share a few features that set them apart from other selenoproteins. They are ER-resident transmembrane proteins with a single transmembrane domain in the N-terminal sequence, contain a Gly-rich segment with positively charged residues, and their Sec residues are characteristically located near the C-terminus. They have been implicated in the ER-associated degradation (ERAD) of misfolded proteins [51]. SELENOK has also been proposed to link selenium levels and immunity through association with a partner enzyme, DHHC6, for protein palmitoylation [109].

### 3.2. Phylogenetic Distribution of Selenoproteins

Selenoproteins are present across the three domains of life: bacteria, archaea, and eukaryotes. The evidence supports that the Sec trait evolved only once: prokaryotes and eukaryotes use analogous Sec biosynthesis and insertion pathways, and some selenoprotein families are shared among bacteria, archaea, and eukaryotes. The use of Sec is not universal, however. In selenoprotein-less organisms, the functions of selenoproteins are typically replaced by Cys homologs and the Sec synthesis machinery genes are lost.

Among prokaryotes, it is estimated that 20–25% of bacteria use selenoproteins [81,110], and the proportion is even lower in archaea, with a narrow distribution of Sec-containing genomes [111]. Nonetheless, the closest relatives to eukaryotes, the archaeal Asgard lineage, was identified as the intermediate form between the prokaryotic and eukaryotic Sec insertion systems [60,112,113].

Sec is much more common among eukaryotes, and selenoproteins show a highly dynamic evolution in terms of Sec to Cys conversions and gene losses. A scattered pattern of the presence/absence of selenoproteins was already evident from the analysis of the early sequenced genomes [114,115]. Since then, thousands of genomes have been sequenced, and the use of bioinformatic tools for large-scale analyses has provided a much more detailed picture. No selenoproteins have been identified in land plants so far, although they are abundant in other Archaeplastida (plantae) lineages. Recent works have explored the diversity of selenoproteins in plantae and especially algae lineages, reporting novel eukaryotic selenoprotein families [116,117]. Fungi were traditionally thought to have lost all selenoproteins at the root of the lineage. This was recently challenged by the discovery of several Sec-containing fungi genomes, outlining multiple independent Sec- loss events, including in the yeast lineage [118]. Among animals, Sec losses have been reported in all major insect lineages [119,120], in mites [34], and in nematodes [121].

## 4. Mouse Models

In the early 2000s, various mouse models were developed to elucidate the role of selenoproteins in health and development [122,123,124,125]. These mouse models took advantage of the fact that selenoprotein expression is dependent on the presence of a single tRNA, Sec tRNA[Ser]^Sec^, and the fact that this tRNA is encoded as a single copy gene, designated Trsp [126]. Thus, by manipulating Trsp in numerous ways, several models were developed that identified the presence of two selenoprotein classes, housekeeping selenoproteins and stress-related selenoproteins, their cellular roles, and also the roles of various individual selenoproteins within these two classes.

### 4.1. Trsp Transgenic Mouse Models

The first mouse models that examined the role of Sec-tRNA[Ser]^Sec^ in selenoprotein synthesis were created in 2001 and involved *Trsp* wild-type or mutant transgenes [92]. Three different constructs encoding either the wild-type or two different mutant transgenes were prepared. By substituting one of the bases within the anticodon loop of *Trsp*, the role of the mutant Sec-tRNA[Ser]^Sec^ in selenoprotein expression could be assessed. Changing either the T at position 34 to A, or the A at position 37 to G, prevented the synthesis of the 2′-0-methyluridine at position 34 [124,127]. Therefore, these mutant mice carrying either mutant transgene permitted the evaluation of the methylribose in selenoprotein expression. Synthesis of stress-related selenoproteins was virtually abolished, while housekeeping selenoprotein expression was virtually unchanged. Stress-related selenoprotein synthesis, therefore, is dependent on the 2′-0-hydroxymethyl group, while housekeeping selenoprotein expression is carried out by Sec-tRNA[Ser]^Sec^_mcm5U_. These studies did not resolve the question of whether Sec-tRNA[Ser]^Sec^_mcm5Um_ also supports housekeeping selenoprotein synthesis. However, it seems very plausible that this isoform can also synthesize the essential class of selenoproteins.

Various aspects of the effects of stress-related selenoprotein loss on health were also examined. Interestingly, reduced stress-related selenoprotein expression in G37 mutant mice was tissue-specific, wherein the loss was highly significant in the kidney and liver but not in the testes [124]. These mice were further studied regarding health issues and were found to be more susceptible to colon cancer [128], viral infection [129], and X-ray damage [130]. Crossing these mice with C3/TAg mice yielded offspring with accelerated rates of prostatic epithelial neoplasia, suggesting a protective role of selenoproteins in prostate cancer development [131]. Glucose intolerance was also observed in these G37 mice, which led to a diabetic-like phenotype [132].

### 4.2. Trsp Conditional Knockout Mouse Models

Although the total removal of *Trsp* in mice is embryonic lethal [122], the targeted removal of *Trsp* in specific tissues and organs provides an alternative model for examining the role of selenoproteins in health and development [123]. Highly significant functions of selenoproteins were elucidated in numerous organs and tissues such as skeletal muscle; heart and endothelial cells [133]; bone [134]; skin [135]; immune cells, including macrophages, hematopoietic tissues, and T cells [136,137,138]; neurons [139]; liver [82,140]; podocytes [141]; osteochondroprogenitor [134]; thyroid [142]; prostate [143]; kidney; and mammary glands [123]. For convenience to the reader, and to keep this review within the allotted length, we have summarized the major findings in each of the above in vivo Trsp conditional knockout studies in Table 3. It should also be noted that more in-depth, recent studies of endothelial cells in cell death have been carried out (see [144] in this Special Issue and references therein).

### 4.3. Trsp Knockout/Transgenic and Trsp Conditional Knockout/Transgenic Mouse Models

Models involving *Trsp* knockout and *Trsp* conditional knockout mice that were rescued with the *Trsp* wild-type transgene, or the G37 or A34 mutant transgenes, were constructed [125] to examine the ability of these transgenes to restore selenoprotein synthesis. As expected, the wild-type transgene completely restored selenoprotein biosynthesis, while the G37 mutant transgene restored housekeeping selenoprotein synthesis but virtually did not refurbish stress-related selenoprotein synthesis [125]. Importantly, these mice demonstrated unequivocally that stress-related selenoproteins are not essential to the livelihood of the animal, although these mice were found to be very susceptible to selenium status (see Table 4). Mice carrying the G37 mutant transgene appeared phenotypically normal, but male mice produced sperm with an abnormal morphology which accounted for their reduced fertility, while female mice produced smaller-sized litters than the corresponding wild-type mice.

The A in the wobble position of the anticodon in tRNA is converted to inosine (I) which decodes A, U, and C in the 3′ position of the corresponding codewords. Hence, this tRNA decodes UGA and the cysteine codons, UCU and UCC, and likely promotes misreading in protein synthesis, which most certainly accounts for the reason why *Trsp* negative mice could not be rescued with the A34 mutant transgene [155].

Additional mouse models lacking *Trsp* specifically in the liver and rescued with the wild-type transgene or G37 or A34 mutant transgenes were generated [82]. A mouse model involving the loss of *Trsp*, and rescued with a transgene carrying a deletion within the activator element, was also prepared [25]. The activator element is required for the binding of the transcription factor, STAF, to transcribe Sec tRNA[Ser]^Sec^ [156]. The major findings of these studies are summarized in Table 4.

## 5. Conclusions

So much of the molecular biology of selenium has been resolved in the past 30 to 40 years (see chapters in [8]) that the selenium field has tapered off considerably. There is still much to be done, primarily in providing more detailed functions of numerous individual selenoproteins and in understanding the roles of LMW selenocompounds in health and development. Herein, we have assembled a variety of topics in the selenium field that involve the unique characteristics of transcribing *Trsp*, determining the primary sequences of Sec-tRNA[Ser]^Sec^_mcm5U_ and tRNA[Ser]^Sec^_mcm5Um_ isoforms and their roles in translating housekeeping and stress-related selenoproteins, discussing numerous aspects of the historical roles of selenium in health and disease, and the molecular biology of selenium and selenoproteins in health and development. We borrowed the title of the classic Italian Western “The Good, the Bad and the Ugly” to include as part of our title as it seemed to perfectly describe the historical roles of selenium in health. This element has its “good” (an essential element in the diet of mammals and many other life forms), its “bad” (the consequences of too little or too much selenium in the diet), and its “ugly” aspects (in some cases, selenium may promote cancer and other health disorders, and extreme selenium deficiency may be lethal). Likewise, selenoproteins also have their “good” (these proteins are responsible in large part for the many health benefits of selenium), their “bad” characteristics (they can promote many health disorders including cancer), and can be “ugly” (loss of several selenoproteins is lethal). It will be of considerable interest to witness the many discoveries in the selenium/selenoprotein field as they continue to unfold in the years to come.

## Figures and Tables

**Table 1:** Sec-tRNA[Ser]^Sec^ isoforms in cultured mammalian cells.

		Sec tRNA[Ser]^Sec^					
			mcm^5^U		mcm^5^Um		
Cell Line	Selenium Supplementation ^a^	% of Total ^b^	%	% of Total ^c^	%	% of Total ^d^	mcm^5^Um/ mcm^5^U ^e^
HL-60	+(chem. defined media)	9.6	38.5	3.70	61.5	5.90	1.60
	−(chem. defined media)	7.5	61.3	4.60	38.7	2.90	0.63
HL-60	+(FBS)	9.4	55.3	5.20	44.7	4.20	0.81
	−(FBS)	7.4	77.0	5.70	23.0	1.70	0.30
CHO	+(FBS)	1.01	45.1	0.46	54.9	0.55	1.22
	−(FBS)	0.86	56.2	0.48	43.8	0.38	0.78
RMT	+(chem. defined media)	1.7	11.8	0.20	88.2	1.50	7.47
	−(chem. defined media)	1.4	35.7	0.50	64.3	0.90	1.80

^a^ FBS: fetal bovine serum; ^b^ percentage of tRNA[Ser]^Sec^ population within total Ser-tRNA population; ^c^ percentages of mcm^5^U and mcm^5^Um isoforms within total tRNA[Ser]^Sec^ population; ^d^ percentages of mcm^5^U or mcm^5^Um isoforms within total Ser-tRNA population; ^e^ amount of mcm^5^Um/amount of mcm^5^U isoforms.

**Table 2:** Sec-tRNA[Ser]^Sec^ isoforms in murine tissues.

		Sec tRNA[Ser]^Sec^					
			mcm^5^U		mcm^5^Um		
Organ	Dietary Selenium Supplementation	% of Total ^a^	%	% of Total ^b^	%	% of Total ^c^	mcm^5^Um/ mcm^5^U ^d^
Heart	+	4.3	38.1	1.64	61.9	2.66	1.62
	−	3.2	66.4	2.12	33.6	1.08	0.51
Kidney	+	7.5	33.7	2.52	66.3	4.97	1.97
	−	3.7	59.2	2.19	40.8	1.51	0.69
Liver	+	4.5	33.3	1.50	66.7	3.00	2.00
	−	2.8	57.7	1.62	42.3	1.18	0.73
Muscle	+	1.9	38.6	0.73	61.4	1.17	1.59
	−	1.5	73.3	1.10	26.7	0.40	0.35

^a^ Percentage of tRNA[Ser]^Sec^ population within total Ser-tRNA population; ^b^ percentages of mcm^5^U and mcm^5^Um isoforms within total tRNA[Ser]^Sec^ population; ^c^ percentages of mcm^5^U or mcm^5^Um isoforms within total Ser-tRNA population; ^d^ amount of mcm^5^Um/amount of mcm^5^U isoforms.

**Table 3:** *Trsp* conditional knockout mouse models.

Targeted Tissue or Organ ^1^	Main Findings Regarding Role of Selenoproteins in Genetically-Altered Mice, Relative to Control Mice in the Study	*Cre* Promoter
Endothelial cells	Endothelial cell development/function: embryonic lethal. 14.5 d.p.c. embryos were smaller, more fragile, had poorly or under-developed vascular systems, limbs, head, and tail [133].	*TieTek2-Cre*
Heart & Skeletal Muscle	Heart disease prevention: mice died from acute myocardial failure 12 days after birth.	*MCK-Cre*
Kidney	No increase in oxidative stress or nephropathy found in podocytes of selenoprotein-deficient mice [141].	*NPHS2-Cre*
Liver	Liver function: severe hepatocellular degeneration—mice died between 1 and 3 months of age [82]. SELENOP and GPX3 were reduced in serum and kidney, supporting a selenium-transport role for liver-derived SELENOP [140]. Enhanced expression of phase II response genes compensated for loss of hepatic *Trsp* [145]. Mice used as controls to monitor selenium pools in kidney due to reduction of GPX3 imported from liver [146]. *Secisbp2* gene inactivation was less detrimental than *Trsp* inactivation [147].	*Alb-Cre*
Macrophages	Immune function: increased oxidative stress and expression of cytoprotective antioxidant and detoxification genes, accumulation of ROS levels, and impaired invasiveness. Altered expression of ECM and fibrosis-associated genes [148]. Balance of pro- and anti-inflammatory oxylipids during inflammation [149]. Selenoproteins protect mice from chemically-induced colitis by alleviating inflammation [150]. Role in epigenetic modulation of pro-inflammatory genes [151]. When infected with *N. brasiliensis*, selenium-supplemented KO mice showed a complete abrogation in M2-marker expression with a significant increase in intestinal worms and fecal eggs [152].	*LysM-Cre*
Mammary glands	First *Trsp* conditional KO mouse, providing an important tool for elucidating the role of selenoproteins in health and development [123]. MMTV-Cre mice treated with DMBA had significantly more tumors, suggesting that selenoproteins protect against carcinogen-induced mammary cancer [153].	*MMTV-Cre; Wap-Cre*
Neurons	Neuronal function: enhanced neuronal excitation followed by neurodegeneration of hippocampus. Cerebellar hypoplasia associated with degeneration of Purkinje and granule cells. Cerebellar interneurons essentially absent [139]. Selenoproteins required in post-mitotic neurons of the developing cerebellum [154].	*Tal-Cre; CamK-Cre*
Osteo-chondroprogenitor	Kashin–Beck disease model: mice had post-natal growth retardation, chondrodysplasia, chondronecrosis, and delayed skeletal ossification characteristic of Kashin–Beck disease [134].	*Col2a1-Cre*
Prostate	Mice developed PIN-like lesions and microinvasive carcinoma by 24 weeks, which were associated with loss of basement membrane, increased cell cycle, and apoptotic activity [143].	*PB-Cre4*
Skin	Role in skin and hair follicle development: runt phenotype, premature death, alopecia with flaky and fragile skin, epidermal hyperplasia with disturbed hair cycle, and an early regression of hair follicles [135].	*K14-Cre*
T-cells	Immune function: reduction of mature T cells and a defect in T-cell-dependent antibody response. Antioxidant hyperproduction and suppression of T cell proliferation in response to T cell receptor stimulation [137].	*LCK-Cre*
Thyroid	Mice lacking selenoproteins in thyrocytes showed increased oxidative stress in thyroid. Gross morphology remained intact for at least 6 months. Thyroid hormone levels remained normal in knockout mice; thyrotropin levels moderately elevated [142].	*Pax8-Cre;* *Tg-Cre^ER^*

^1^ Target organs/tissues in alphabetical order. Abbreviations: days-post-coitum (d.p.c.); 7,12-dimethylbenz[a]anthracene (DMBA); extracellular matrix (ECM); mouse mammary tumor virus (MMTV); prostatic intraepithelial neoplasia (PIN).

**Table 4:** Mouse models involving Trsp knockout (KO) or Trsp conditional KO mice rescued with wild-type (WT), G37 mutant, or A34 transgenes.

Target Site	Model Description	Major Findings Observed in Genetically Altered Mice in Comparison to Control Mice
Whole Mouse	*Trsp* KO rescued with WT *Trsp* transgene	Selenoprotein synthesis was completely recovered [125].
	*Trsp* KO rescued with G37 *Trsp* transgene	Proper base modification in the anticodon is essential, as mutant mice synthesize stress-related selenoproteins very poorly. Male mutant mice show abnormal sperm and reduced fertility; females produced reduced litter size [43]. Trsp KO could not be rescued with A34 mutant transgene most likely due to misreading (see Text).
Whole Mouse	*Trsp* KO rescued with promoter mutant *Trsp* transgene	Mice expressed tissue- and organ-specific amounts of tRNA[Ser]^Sec^. Lower levels of the mcm^5^Um isoform were observed in promoter mutant *Trsp* mice. Mice developed a similar neurological phenotype as SELENOP-KO mice and a reduced life span [157].
Liver *Alb-Cre*	*Trsp* liver KO rescued with *Trsp* WT transgene	Selenoprotein synthesis was completely recovered [82].
	*Trsp* liver KO rescued with G37 mutant *Trsp* transgene	Housekeeping selenoprotein synthesis was recovered while stress-related selenoprotein synthesis was poorly recovered [82].
	*Trsp* liver KO rescued with A34 mutant *Trsp* transgene	Housekeeping selenoprotein synthesis was recovered while stress-related selenoprotein synthesis was poorly recovered. Replacement of selenoprotein synthesis in conditional Trsp mutants resulted in normal gene expression of Phase II response enzymes [127,145].

## Data Availability

Not applicable.
